# Social isolation stress facilitates chemically induced oral carcinogenesis

**DOI:** 10.1371/journal.pone.0245190

**Published:** 2021-01-07

**Authors:** Flávia Alves Verza, Vitor Bonetti Valente, Lia Kobayashi Oliveira, Giseli Mitsuy Kayahara, Marcelo Macedo Crivelini, Cristiane Furuse, Éder Ricardo Biasoli, Glauco Issamu Miyahara, Sandra Helena Penha Oliveira, Daniel Galera Bernabé

**Affiliations:** 1 Laboratory of Psychoneuroimmunology, Psychosomatic Research Center and Oral Oncology Center, São Paulo State University (UNESP), School of Dentistry, Araçatuba, São Paulo, Brazil; 2 Department of Diagnosis and Surgery, School of Dentistry, São Paulo State University (UNESP), Araçatuba, São Paulo, Brazil; 3 Department of Basic Sciences, Laboratory of Immunopharmacology, School of Dentistry, São Paulo State University (UNESP), Araçatuba, São Paulo, Brazil; University of Toronto, CANADA

## Abstract

Social isolation has affected a large number of people and may lead to impairment of physical and mental health. Although stress resulting from social isolation may increase cancer progression, its interference on tumorigenesis is poorly known. In this study, we used a preclinical model to evaluate the effects of social isolation stress on chemically induced oral carcinogenesis. Sixty-two 21-day-old male Wistar rats were divided into isolated and grouped groups. After 90 days of age, the rats from both groups underwent oral carcinogenesis with 4-nitroquinoline 1-oxide (4NQO) for 20 weeks. All rats were assessed for depressive-like behavior and euthanized for oral squamous cell carcinoma (OSCC) diagnosis and measurement of inflammatory mediators in the tumor microenvironment. Social isolation stress increased the OSCC occurrence by 20.4% when compared to control. Isolated rats also showed higher tumor volume and cachexia than the grouped rats. Social isolation did not induce changes in the depressive-like behavior after carcinogenic induction. Tumors from stressed rats had increased levels of the inflammatory mediators, TNF-alpha, IL1-beta and MCP-1. The concentrations of TNF-alpha and MCP-1 were significantly increased in the large tumors from isolated animals. Higher tumor levels of TNF-alpha, IL-6, IL1-beta and MCP-1 were positively correlated with OSCC growth. This study provides the first evidence that social isolation stress may facilitate OSCC occurrence and tumor progression, an event accompanied by increased local levels of inflammatory mediators.

## Introduction

Currently, several people worldwide are isolating during the COVID-19 pandemic [1]. This disruption of interactions between individuals may trigger psychological and neuroendocrine responses that could increase the incidence, morbidity and mortality of a great number of diseases [2–5]. People with strong social relationships can have a survival rate 50% higher than the isolated individuals [4]. Social isolation has been widely considered a potent stressor characterized by the lack of social contact, such as family and friends [2,3]. However, it does not affect only people who live alone once the feeling of loneliness can also impact individuals who reside with other people [6].

A growing number of studies have been investigating the association between emotional disorders and cancer progression [7–10]. Social isolation can influence cancer progression by altering gene expression [11], tumor angiogenesis [12], behavior [13], neuroendocrine [13–15] and immune responses [14,16]. In preclinical models of breast [14,15] and liver cancer [16], social isolation accelerates tumor growth. Social isolation and lack of social support may decrease survival rate in cancer patients [17,18]. Cancer progression has been associated with stress-mediated modulation of pro-inflammatory cytokines such as TNF-α, IL-6 and IL1-beta [19]. These mediators released in the tumor microenvironment, as consequence of stressful social conditions, can contribute to tumor growth, angiogenesis and metastasis [20]. Social disruption also promotes the monocyte chemoattractant protein-1 (MCP-1) secretion, a chemokine produced by the inflammatory cells [21]. Chronic stress can modulate the MCP-1 levels into the microenvironment and increase tumor growth [22].

Oral squamous cell carcinoma (OSCC) is the most common type of head and neck cancer and occurs frequently in elderly men [23,24]. The main risk factors for OSCC occurrence are long-term tobacco use and alcohol consumption [25]. Although studies have analyzed the impact of chronic stress or stress-related mediators on OSCC progression [26–29], the effects of social isolation on OSCC occurrence and progression have not been evaluated. Thus, we hypothesized that social isolation stress could facilitate chemically induced oral carcinogenesis and tumor progression. In the present study, a well-established preclinical model of oral carcinogenesis was used to investigate the effect of social isolation on the occurrence and progression of chemically induced OSCCs. Levels of inflammatory mediators were evaluated in the tumor microenvironment. Furthermore, depressive-like behaviors were investigated after oral carcinogenesis induction.

## Materials and methods

### 2.1 Animals and experimental conditions

All experiments with animals followed the National Institutes of Health Guide for the Care and Use of Laboratory Animals and were approved by the Institutional Animal Welfare Committee at the São Paulo State University (UNESP), School of Dentistry, Araçatuba, São Paulo. Sixty-two male Wistar rats were obtained from the Central Animal Care Facility of the university and housed in ventilated cages, which were placed in an environment room with controlled temperature (25±2°C), humidity (55±5%) and light (12-h light/dark cycle). The animals had *ad libitum* access to drinking water and standard pellet diet (Purina, Paulínia, SP, Brazil) throughout out the experimental period.

### 2.2 Experimental design

**Fig 1:**
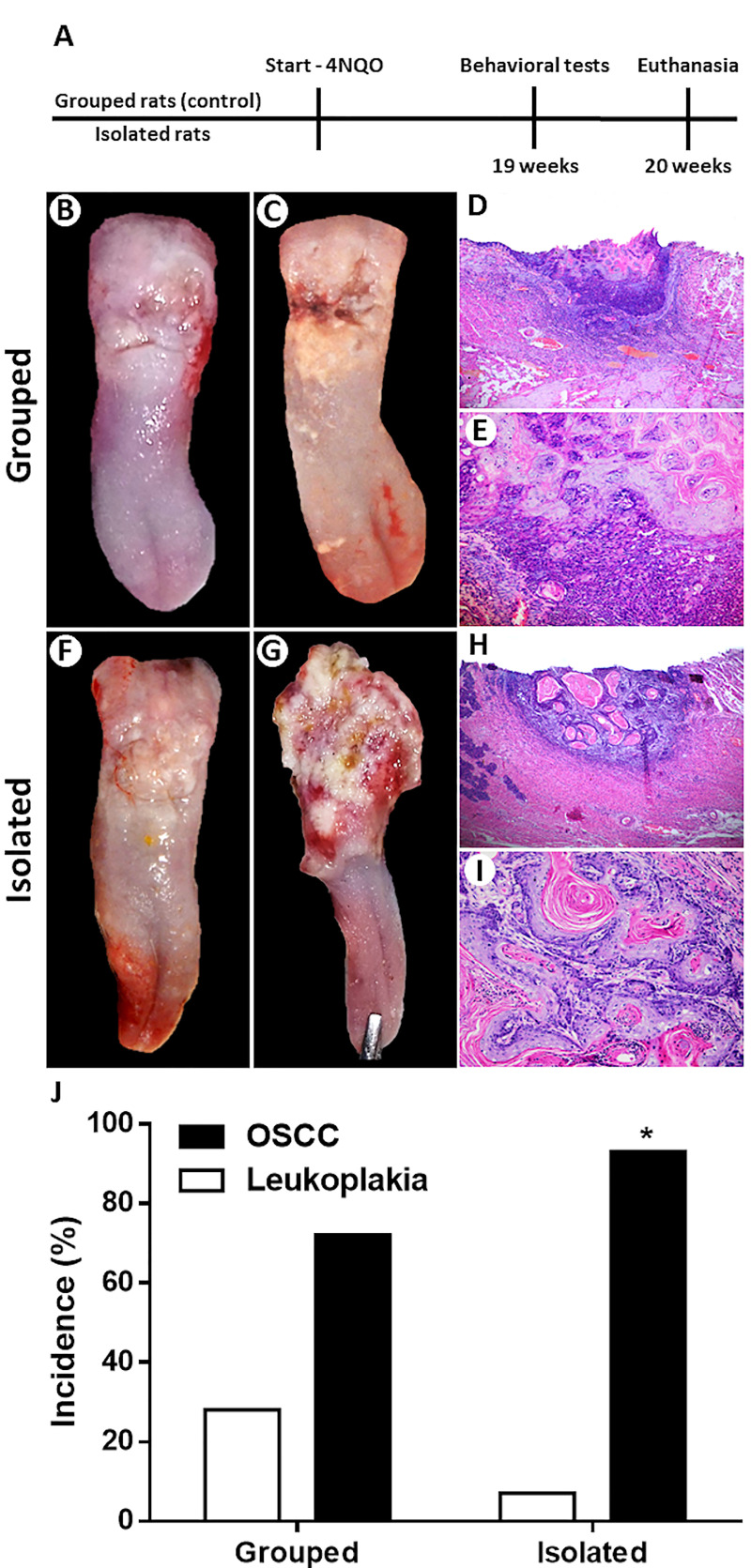
Experimental design **(A)**. Clinicopathological characteristics of the OSCCs from grouped rats **(B-E)**. White plates with a small ulcer **(B)**. Yellowish white plaques with an infiltrative ulcer **(C)**. Small-size OSCC (H&E; 50x) **(D)**. Tumor epithelial cells surrounded by a chronic inflammatory infiltrate (H&E; 200x) **(E)**. Clinicopathological features of the OSCCs from isolated rats **(F-I)**. Irregular yellowish white plates **(F)**. Extensive ulcer with reddish-white areas **(G)**. Large-size OSCC (H&E; 50x) (H). Well-differentiated cells arranged in islands of varying size containing keratin pearls (H&E; 200x) **(I)**. Social isolation stress increased the occurrence of chemically induced OSCC **(J)**. Chi-square test showed that isolated rats had higher occurrence of OSCC than control rats. *p<0.05. Bar graphs represent the incidence rate of leukoplakia and OSCC for both groups.

The study was conducted with two experimental groups: 1) Isolated group: 30 rats subjected to social isolation stress (individually conditioned animals); 2) Grouped group: 32 rats kept in groups of four animals per cage. After reaching 90 days of age, rats from both groups were subjected to oral carcinogenesis for 20 weeks. The animals underwent to behavioral tests one week before the end of carcinogenic induction. Histopathological analysis was performed to assess OSCC occurrence and tumor invasion. Levels of pro-inflammatory cytokines TNF-alpha, IL-6 and IL1-beta and the monocyte chemoattractant protein-1 (MCP-1) concentrations were analyzed in the OSCC microenvironment. Fig 1A illustrates the experimental design of the study.

### 2.3 Social isolation stress model

On the 21^st^ postnatal day, the rats from isolated group were separated from their mothers. This age was chosen to initiate isolation because it is critical for the development of social behavior [11–13]. The rats were then kept isolated (one animal per cage) in polypropylene boxes measuring 30 x 19 x 13 cm lined with shavings [11,14–16]. Social isolation stress was maintained until the end of experimental period. The grouped rats (control group) were also separated from the mothers on the 21st postnatal day. They were kept in polypropylene boxes measuring 41 x 34 x 16 cm lined with shavings during the experimental period.

### 2.4 Depressive-like behavior tests

The tail suspension and forced swimming tests were used to assess the depressive-like behavior of rats from both groups. Both tests are based on the same principle: the rats are exposed to an unavoidable situation [30]. *Tail suspension test (TST)*. The TST was performed using a box measuring 32 × 33 × 33 cm with an open front side that allowed visual observation during the test. The rats were individually suspended by the tail for 6 minutes using an adhesive tape placed 2.5 cm from the tail tip. *Forced swimming test (FST)*. The FST was performed 7 days after TST. The test was divided into two sessions. In the first session (pre-test), the rats were forced to swim for 15 minutes in a narrow cylinder (13 cm in diameter × 24 cm in height) containing water (25°C ± 2°C). Pre-test was performed to adapt the rats for the test itself. The second session (test itself) was performed 24 hours after the pre-test. In this session, the rats were submitted to the forced swimming test for 5 minutes under the same pre-test conditions. Both behavioral tests were performed one week before euthanasia. The rats were videotaped during the tests for later analysis. Two researchers (FAV and LKO) who were blinded to the experimental groups analyzed the videos. The immobility times of 15 grouped rats and 14 isolated rats randomly chosen were used to determine the depressive-like behavior in both tests. The average times of immobility measured by FAV (observer 1) and LKO (observer 2) for the TST and FST tests were highly correlated with each other and the data obtained by observer 1 were used to represent the results of these assays.

### 2.5 Oral carcinogenesis model

For OSCC induction, all rats from two groups were treated with 50 ppm of 4-nitroquinoline-1-oxide (4NQO) (Lot Number # WXBC3635V, Sigma-Aldrich, St. Louis, USA) solution diluted in the drinking water. In this model, OSCC develops mainly in the dorso-posterior region of the tongue [31]. The rats had free access to water with carcinogen for 20 weeks [29,31]. After this period, all rats were euthanized by decapitation and their tongues with the carcinogen-induced lesions were removed. Then, the tongues were sectioned longitudinally into two halves to perform the histopathological analysis and measurement of inflammatory mediators.

### 2.6 Histopathological analysis

Half of the tongues were then fixed in 10% buffered formaldehyde for 48 hours, embedded in paraffin blocks, sectioned at 4 μm and stained with hematoxylin and eosin (H&E). The tongue lesions derived from carcinogen treatment were classified in leukoplakia and oral squamous cell carcinoma (OSCC) according to WHO criteria [32]. Leukoplakia is considered a precursor lesion for the development of OSCC [33]. The following microscopic features were analyzed for the diagnosis of leukoplakia: atrophy of the epithelium, hyperkeratosis, hyperplasia and epithelial dysplasia (mild, moderate and severe) [34]. The Binary Grading System was used for grading oral epithelial dysplasia as low- or high-risk lesions [35]. According to Bryne’s criteria [36], the degree of tumor malignancy was evaluated in the OSCC invasive front by grading the following microscopic features: keratinization degree, nuclear pleomorphism, pattern of invasion and inflammatory infiltrate. Two oral pathologists (MMC and CF) who were blinded to the experimental groups assessed individually the histopathological features of leukoplakias and OSCCs. The data obtained by each pathologist were correlated to each other and the differences were resolved by consensus.

### 2.7 Tumor volume and body weight variation

The tumor samples from isolated and grouped rats collected after euthanasia were used to calculate the tumor volume. The three major dimensions (depth, width and length) of the tumors were obtained in millimeters as previously described [29]. Tumor volume was calculated in mm^3^ using the formula: volume = depth x width x length [29]. Body weight of each animal was recorded weekly. After carcinogenic induction, the weight variation was calculated in grams using the measurements obtained one day before starting 4NQO treatment and one day before euthanasia.

### 2.8. Measurement of inflammatory mediators

#### 2.8.1. Sample preparation

For measuring the levels of pro-inflammatory cytokines TNF-alpha, IL-6 and IL1-beta and the chemokine monocyte chemoattractant protein-1 (MCP-1) in the tumor microenvironment, OSCCs samples (2 x 2 x 2 mm) obtained from other half of the tongues were homogenized in PBS buffer with a cocktail protease inhibitor (Roche Diag., Mannheim, Germany). The tissue homogenates were centrifuged for 5 min at 13.000 rpm and the supernatants aliquoted and stored at -80°C until use.

#### 2.8.2. Analysis of TNF-alpha, IL-6, IL1-beta and MCP-1 concentrations

The concentrations of TNF-alpha, IL-6, IL1-beta and MCP-1 in the supernatants were measured 72 hours after euthanasia by the Milliplex Multiple Analyte Profiling method through the Luminex Corporation’s xMAP™ technology. To perform the assay, a Milliplex™ kit (TNF-alpha, IL-6, IL1-beta and MCP-1, Catalog. No: # RADPCMAG-82K-05; Millipore, St. Charles, Missouri, USA) was used according to the manufacturer's recommendations. The assay sensitivities were 0.1 pg/mL for TNF-alpha, 11 pg/mL for IL-6 and 0.9 pg/mL for IL1-beta. For all targets, the intra- and inter-assays CVs were <10% and <15%, respectively. The assay was run in triplicate.

#### 2.8.3. Normalization of the data

Total protein concentration was measured in the tissue supernatants according to Lowry et al. [37] and the data were used to normalize the levels of each inflammatory mediator. The concentrations of mediators were expressed as pg (of mediator)/μg (of total protein) in 1 mL of tissue supernatant.

### 2.9 Statistical analysis

GraphPad Prism version 8.2 (GraphPad Software Inc., Dan Diego, CA, USA) and SAS version 9.3 (SAS Institute, Cary, NC, USA) were used to perform the statistical tests. Chi-square test evaluated whether social isolation stress was associated with OSCC occurrence and the degree of tumor malignancy. The data distribution of clinical features, depressive-like behaviors and levels of inflammatory mediators in the tumor microenvironment was assessed by the Shapiro-Wilk test. In presence of normality, the mean comparisons were performed by the Student’s test-t or analysis of variance test (ANOVA), followed by the Tukey multiple comparison test to determine whether there were differences between the groups. In absence of normality, the mean comparisons were performed by adjusting the Gamma distribution of the data, followed by Wald's multiple comparison test. Pearson correlation test was used to assess the associations between the levels of TNF-alpha, IL-6, IL1-beta and MCP-1 and tumor volume. The statistical significance was considered at a *p* value less than 0.05 (p<0.05) for all tests.

## Results

### 3.1. Social isolation stress increases the risk of chemically induced oral cancer

Firstly, we investigated the effects of social isolation on the OSCC occurrence in male Wistar rats underwent chemically induced carcinogenesis. Three rats from control group and two rats from isolated group were sensitive to the toxic effects of 4NQO treatment and died during the first month of oral carcinogenesis. There was no significant difference in 4NQO carcinogen consumption between the two experimental groups (p> 0.05; data not shown). All rats developed single lesions in the dorso-posterior region of the tongue. The clinicopathological characteristics of the tumors from grouped and isolated rats can be seen in Fig 1B–1I. Among the grouped rats, 21 rats (72.4%) developed OSCC while 8 rats (27.6%) had leukoplakia (1—hyperkeratosis, 1—hyperplasia, 3—moderate dysplasia, 3—severe dysplasia). In the stressed group, 26 rats (92.8%) developed OSCC and only 2 rats (7.2%) had leukoplakia (1—hyperplasia, 1- severe dysplasia) after 20 weeks of chemically induced carcinogenesis. There was an increase of 20.4% in the OSCC occurrence in the isolated group compared to control group (p = 0.002; Fig 1J).

### 3.2. Social isolation enhances tumor volume and promotes cachexia

**Fig 2:**
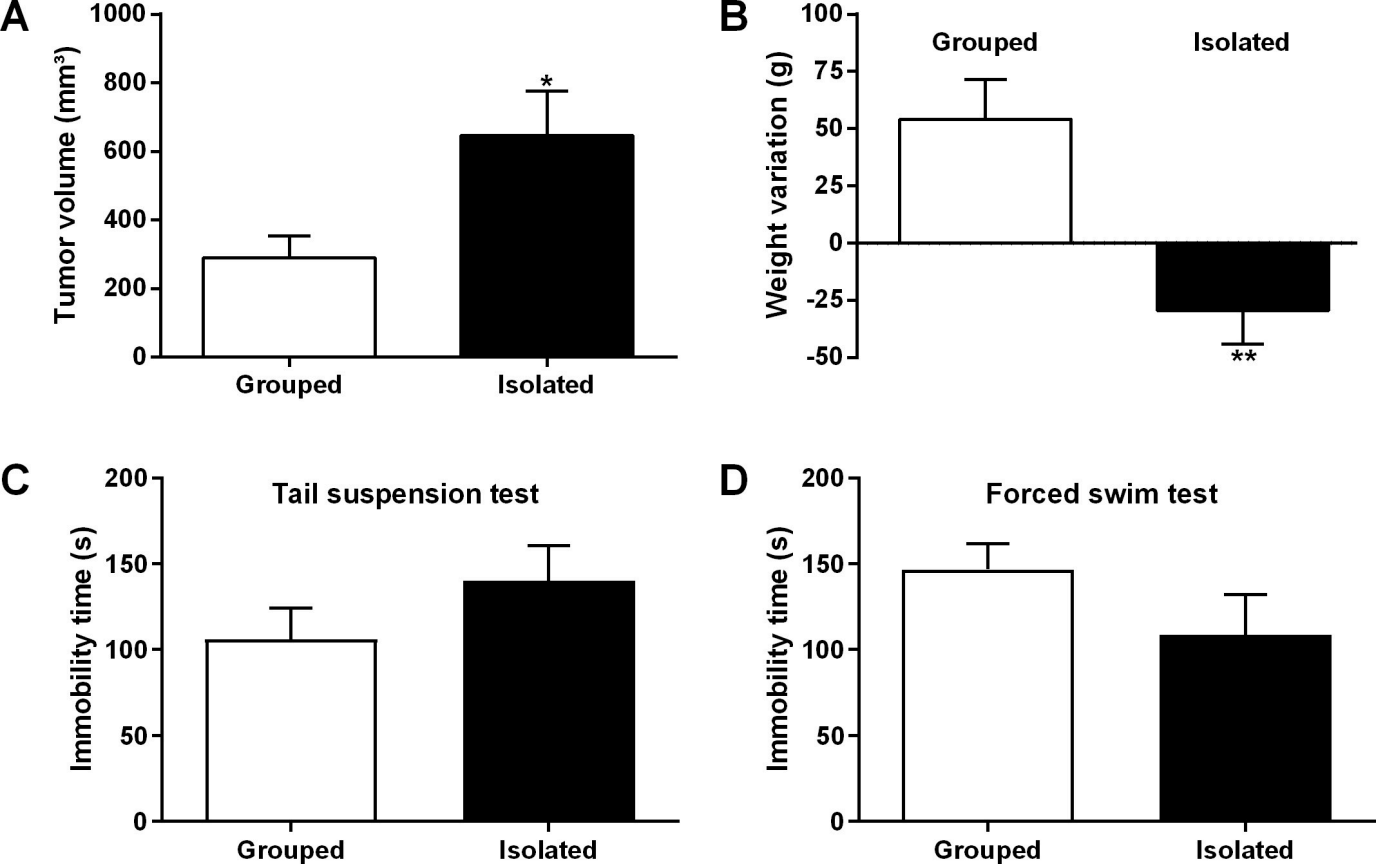
Social isolation stress promotes tumor growth and weight loss in OSCC rats. Isolated rats (n = 26) had increased tumor volume compared to grouped rats (n = 21) **(A)**. Isolated animals (n = 26) showed weight loss while grouped rats (n = 21) exhibited body weight gain after carcinogen treatment **(B)**. Social isolation stress did not affect the depression-like behavior in cancer rats (grouped, n = 15; isolated, n = 14). There were no significant differences between the groups in the immobility time measured by TST **(C)** (p>0.05) and FST **(D)** (p>0.05). Student’s teste T; *p<0.05 and **p<0.001. Bar graphs represent mean ± standard error of the mean (SEM).

The results showed that rats subjected to social isolation stress had a higher tumor volume (646.8 ± 180.7 mm^3^) when compared to grouped animals (290.3 ± 105 mm^3^) (p = 0.0328; Fig 2A). Isolated animals displayed tumors approximately two-fold larger than the rats from control group (Fig 2A). Isolated rats also had loss of body weight (-29.46 ± 15.83 g) while grouped rats displayed weight gain (54.24 ± 21.28 g) (p = 0.0004; Fig 2B) after the carcinogenic induction. According to Bryne’s criteria [36], most OSCCs exhibited low degree of malignancy (isolated, 76.2% vs grouped, 66.6%; p = 0.5237). In both groups, most of OSCCs showed a high degree of keratinization (isolated, 54.5%; grouped, 55%), little nuclear pleomorphism (isolated, 50%; grouped, 65%), well-delineated borders (isolated, 40%; grouped, 60%) and marked inflammatory infiltration (isolated, 63.6%; grouped, 55%). There was no statistical difference between the groups regarding the Bryne’s criteria (p>0.05). Most leukoplakias were categorized as high-risk dysplasias (isolated, 50% vs grouped, 37.5%; p = 0.6592) according to the Binary Grading System [35].

### 3.3. Social isolation does not affect depressive-like behavior in rats with oral cancer

In order to evaluate whether social isolation stress affects depressive behaviors in rats with OSCC, TST and FST were performed after carcinogenic induction. There were no differences between isolated and control rats regarding the depressive-like behaviors (p>0.05; Fig 2C and 2D). In the TST, the immobility time from isolated rats (140 ± 33.94 s) was slightly higher when compared to grouped animals (106 ± 27.43 s) (Fig 2C). On the other hand, in the FST, the immobility time of isolated rats (108.5 ± 38.30 s) was slightly decreased when compared to grouped rats (146.8 ± 27.69 s) (Fig 2D). In both tests, the results were not statistically significant (p = 0.2249 and p = 0.1780, respectively).

### 3.4. Tumors from isolated rats display increased levels of inflammatory mediators

**Fig 3:**
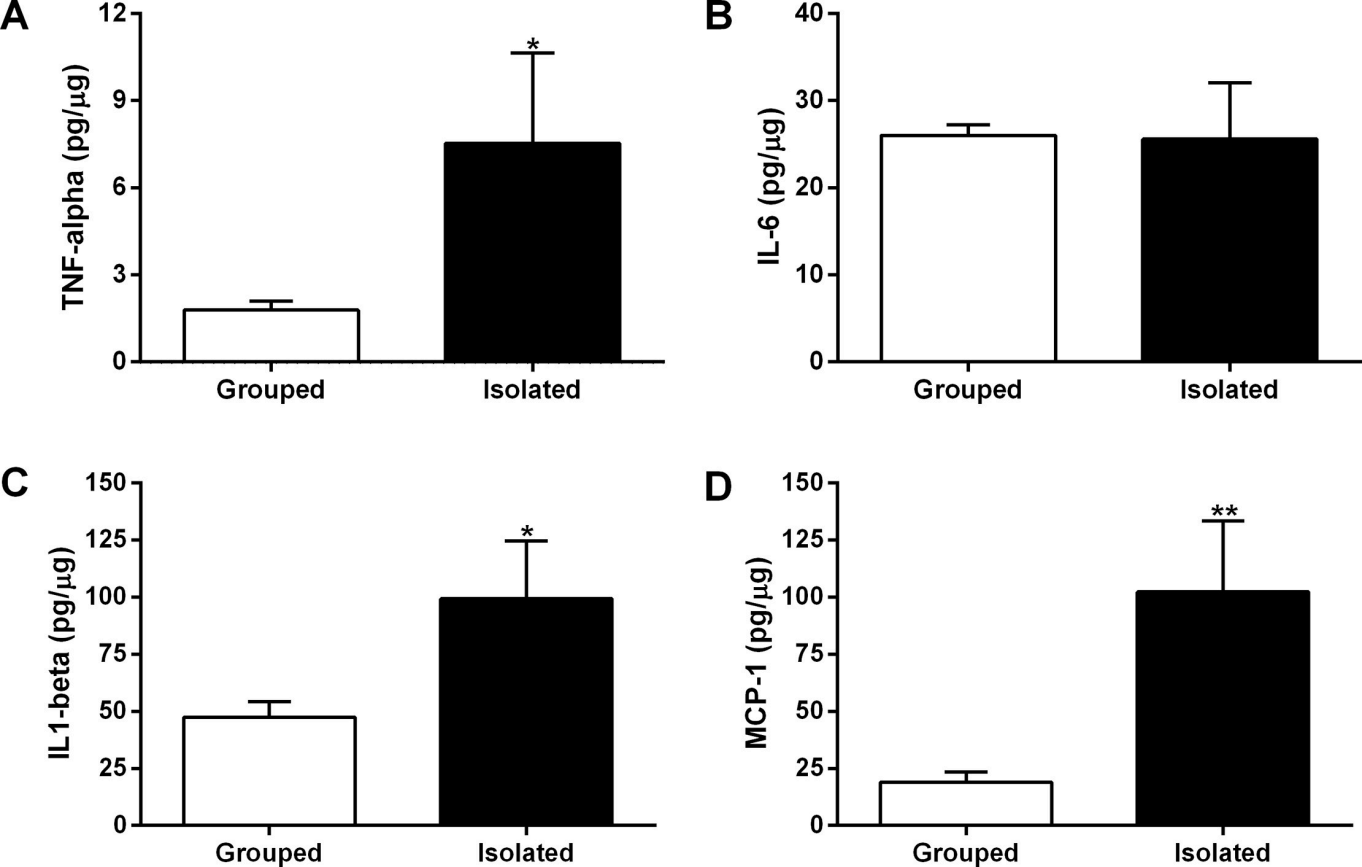
Levels of inflammatory mediators in the OSCC microenvironment. TNF-alpha **(A)**, IL-6 **(B)**, IL1-beta **(C)** and MCP-1 levels **(D)**. Eight tumor samples from each group were randomly chosen to perform the analyzes. Student’s test T displayed that the levels of inflammatory mediators, except the IL-6 levels, were increased in the tumor microenvironment from isolated animals. *p<0.05 and **p<0.001 indicate a statistically significant difference. Bar graphs represent the mean ± standard error of the mean (SEM).

Milliplex Multiple Analyte Profiling assay was performed to assess whether social isolation stress induced changes in the levels of inflammatory mediators related to OSCC progression (Fig 3A–3D). The levels of TNF-alpha and IL1-beta and MCP-1 were increased in the tumor microenvironment from isolated rats (p<0.05; Fig 3A, 3C and 3D). TNF-alpha levels were about 4-fold higher in the tumor microenvironment from stressed rats (isolated, 7.531 ± 3.099 pg/μg vs grouped, 1.787 ± 0.3129 pg/μg) (p = 0.0034; Fig 3A). IL1-beta levels were two times higher in the OSCCs from isolated rats (isolated, 99.33 ± 25.24 pg/μg) than in the tumors from grouped rats (47.39 ± 6.835 pg/μg) (p = 0.0149; Fig 3C). MCP-1 levels were approximately 5-fold higher in the OSCCs from isolated rats (102.3 ± 31.04 pg/μg) compared to tumors from control rats (19.12 ± 4.465 pg/μg) (p = 0.0009; Fig 3D). There was no significant difference between groups concerning the IL-6 concentrations in the tumor microenvironment (isolated, 25.57 ± 6.479 pg/μg vs grouped, 26.01 ± 1.241 pg/μg) (p = 0.9360; Fig 3B).

**Fig 4:**
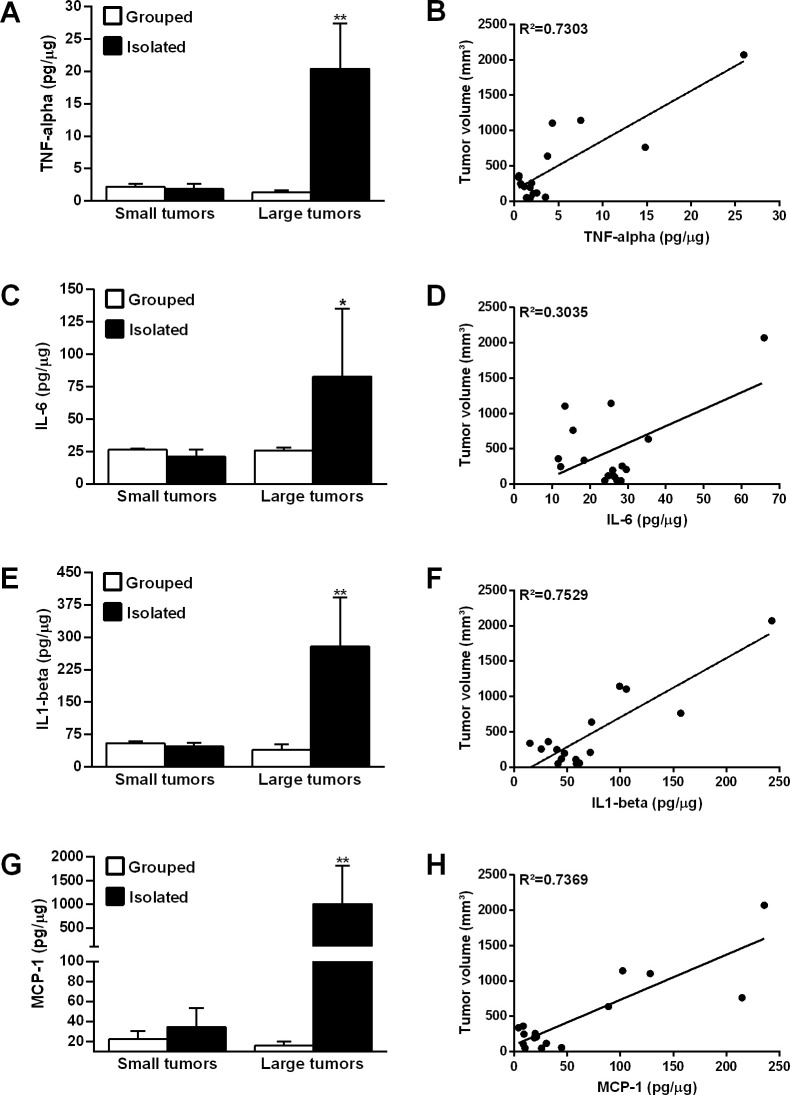
Levels of the tumor inflammatory mediators according the OSCC stage. TNF-alpha **(A)**, IL-6 **(C)**, IL1-beta **(E)** and MCP-1 levels **(G)**. ANOVA followed by Tukey multiple comparison demonstrated that the levels of inflammatory mediators were increased in the large tumors from isolated rats. Correlation between levels of the inflammatory mediators and OSCC volume. The Pearson correlation test showed a positive relationship between the tumor levels of TNF-alpha **(B)**, IL-6 **(D)**, IL1-beta **(F)** and MCP-1 **(H)** and tumor volume. *p<0.05 indicates a statistically significant difference. *p<0.05 and **p<0.001. Bar graphs represent the mean ± standard error of the mean (SEM).

To assess the association between tumor size and the levels of inflammatory mediators in the tumor microenvironment, OSCCs from both groups were classified into small and large sizes according to the median split of the tumor volume (Fig 4) [38]. Levels of inflammatory mediators were significantly higher in large OSCCs from isolated rats than in large tumors from control rats (p<0.05; Fig 4A, 4C, 4E and 4G). Among the large tumors, TNF-alpha levels were about 15 times higher in OSCCs from isolated rats (20.42 ± 6.964 pg/μg) compared to tumors from grouped rats (grouped, 1.351 ± 0.3352 pg/μg) (p<0.0001; Fig 4A). The concentrations of IL-6 (isolated, 82.65 ± 52.45 pg/μg vs grouped, 25.64 ± 2.503 pg/μg; p = 0.014; Fig 4C) and IL1-beta (isolated, 278.8 ± 113.8 pg/μg vs grouped, 39.86 ± 12.61 pg/μg; p<0.0001; Fig 4E) were, respectively, 3- and 7-fold higher in large tumors from isolated rats. MCP-1 levels were about 63 times higher in large OSCCs from isolated rats (isolated, 1007 ± 814 pg/μg) compared to control rats (15.99 ± 3.99 pg/μg) (p<0.001; Fig 4G). No significant difference was found between groups when the levels of inflammatory mediators were analyzed in the small size tumors (p>0.05; Fig 4A, 4C, 4E and 4G).

### 3.5. Levels of inflammatory mediators in the tumor microenvironment are associated with cancer stage

Correlation tests were performed in order to evaluate whether the concentrations of inflammatory mediators in the OSCC microenvironment were associated to the tumor volume (Fig 4B, 4D, 4F and 4H). The levels of TNF-alpha (p<0.0001; Fig 4B), IL-6 (p = 0.027; Fig 4D), IL1-beta (p<0.0001; Fig 4F) and MCP-1 (p<0.0001; Fig 4H) in the tumor microenvironment were positively correlated with OSCC volume.

## Discussion

The current study promotes the first evidence that social isolation stress may accelerate the onset and progression of OSCC in rats subjected to chemically induced carcinogenesis. Isolated rats displayed cachexia and increased levels of inflammatory mediators in the tumor microenvironment. Social isolation stress has been associated to tumor progression in preclinical models of liver [16] and breast cancer [11,14,15]. Our findings show for the first time that the stress resulting from social isolation can also increase the risk of developing head and neck cancer in a preclinical model. In humans, social isolation has been linked to accelerated cancer progression and a higher risk of disease mortality [17,18].

Our results demonstrated that isolated rats had higher OSCC occurrence induced by 4NQO than the grouped animals. This carcinogen can promote the development of a variable number of lesions in oral cavity, oropharynx and esophagus. Our study found carcinogen-induced lesions only in dorso-posterior region of the tongue. No lesions were found in other regions of the oral cavity or in oropharynx and esophagus. So far, only one study has evaluated the impact of chronic stress on oral carcinogenesis in animals [27]. In rats, Rivera et al. [27] showed that restraint stress did not increase the occurrence and severity of chemically induced OSCCs using the same 4NQO-induced oral carcinogenesis model. In another study, Xie et al. [28] showed that restraint stress induced OSCC growth. However, in this study, the effects of restraint stress were tested using an orthotopic model of OSCC progression in nude mice [28]. The distinct results obtained by Rivera et al. [27] and us can indicate that the effects of chronic stress on chemically induced oral carcinogenesis may be related to the type of stress and the age at which it is induced. Social isolation and restraint stress differently impact biological responses in cancer animals such as adaptive immunity and tumor angiogenesis [39]. In the present study, social isolation stress started in the post-weaning period, whereas in the previous investigations restraint stress was carried out in adulthood [27,28]. Biological negative effects derived from social isolation stress may occur since post-weaning period to adulthood [40–42]. Rat pups that do not develop in a social environment, for example, tend to be more anxious during juvenile stage [40] and adulthood [41]. In cancer, social isolation increases the expression of genes encoding metabolic pathway enzymes promoting tumor development [11]. Furthermore, this lack of social support is associated with the increased activity of beta-adrenergically-linked transcription pathways, which are related to the pathogenesis of disease [43]. Our results suggest that the effects of social isolation stress on oral carcinogenesis would be more significant when the animals are subjected to stressful conditions in the early stages of life.

Animals subjected to social isolation since weaning may display a heightened stress response accompanied by the increased release of corticosterone in the serum [11]. This upregulation has been associated to changes in gene expression and tumor progression [11]. In local level, 4NQO treatment significantly increases the levels of corticosterone in the OSCC microenvironment [29]. Although we did not measure the glucocorticoid levels, higher systemic and/or local levels of corticosterone may have contributed for greater progression of OSCC in the isolated rats of the current study. Here, isolated rats displayed OSCCs approximately 2-fold higher than grouped rats. Likewise, preclinical studies with other types of cancer have also shown similar results. Isolated female mice developed chemically induced breast carcinomas with greater tumor volume when compared to the tumors from grouped animals [14]. In male Balb/c mice, Wu et al. [12] showed that social isolation induced an increased growth of colon carcinoma cells after intrahepatic implantation. In another study, isolated female mice had greater mammary gland tumors than grouped mice [11]. Clinical evidences have also revealed that the lack of social support may affect cancer progression [44]. Patients with head and neck cancer who are married have lower occurrence of regional metastasis and better survival than unmarried ones [45,46]. Although previous studies have shown that isolated animals did not lose weight after tumor induction [13,47], our results demonstrated that isolated cancer rats lost body weight when compared to grouped OSCC animals. The presence of the tumor may have affected the food intake of the isolated OSCC rats that in turn showed larger tumors when compared to control rats. Furthermore, this loss of body weight may be derived from the tumor-induced cachexia [48]. Tumor-induced cachexia achieves up to 80% of the advanced-stage cancer patients, including those with head and neck cancer malignancies [49]. These patients exhibit significant weight loss and some of them report concerns about eating, especially when receiving palliative care [49]. Although the food consumption during carcinogenesis period was not measured in the current study, isolated rats could have lost body weight due to a lower food intake and by losing adipose tissue and skeletal muscle mass, required in the increased metabolic waste [48].

Increased depressive-like behavior is one of the main consequences of chronic stress derived from social isolation [50–52]. In the current study, social isolation did not affect the depressive-like behavior of cancer rats, even though these animals have showed a higher tumor volume. In other preclinical models without cancer induction, isolated animals showed a more depressive-like behavior than controls [53,54]. In these studies, social isolation stress was induced in adolescence or adulthood after the animals had lived in groups. Here, the rats were isolated from post-weaning. Pisu et al. [55] found a less depressive behavior in isolated rats when social isolation stress started in the post-weaning. In another study, Lamkin et al. [56] assessed the effects of tumor on depressive-like behavior in a mice ovarian cancer model. Similarly to our findings with oral cancer, there was no significant effect of the ovarian tumor on TST immobility time [56]. A limitation of the present study is that the rats of both groups were not evaluated at baseline for depressive-like behavior. Changes induced by the 4NQO carcinogen in brain regions associated to anxiety and depression are still unknown. In the current study, these changes could mask the depressive behavior induced by the social isolation stress in cancer rats. Further studies are needed to investigate mechanisms involved in the 4NQO-induced experimental carcinogenesis that can be associated to depressive-like behavior.

Chronic stress may promote cancer progression through an enhanced tissue expression of tumor progression-related molecules [57]. In the present study, social isolation stress was also associated to an increased concentration of inflammatory mediators in the tumor microenvironment. The levels of TNF-alpha, IL1-beta and MCP-1 were increased in the OSCCs samples from isolated animals. In rats of both sexes, the deprivation of the social contact since weaning may elevate the systemic levels of TNF-alpha, IL-6 and IL1-beta and lead to metabolic abnormalities [58]. Experimental and clinical studies have found a significant relationship between social factors and increased levels of tumor progression-related mediators [12,59]. In male mice with intrahepatic implantation of colon 26-L5 tumor, social isolation stress increased the TNF-alpha levels in the tumor microenvironment [12]. In the current study, OSCCs from isolated rats showed about 4-fold higher levels of TNF-alpha than the tumors from grouped rats. Furthermore, MCP-1 concentrations were about 5-fold higher in the OSCC microenvironment from isolated rats. Similarly, Armaiz-Pena et al. [22] observed that increased MCP-1 levels elevated macrophage recruitment and ovarian cancer growth in nude mice subjected to chronic restraint stress. In this research, peripheral blood monocytes and tumor-associated macrophages were related to worse overall survival in ovarian cancer patients [22]. In patients with oral cancer, local and systemic higher levels of pro-inflammatory cytokines TNF-alpha, IL-6 and IL1-beta may contribute to tumor progression leading to a poor prognosis of the disease [60,61]. In the current study, tumor concentrations of TNF-alpha, IL-6, IL1-beta and MCP-1 were positively correlated with a higher OSCC volume. Together, our findings suggest that social isolation stress may potentiate head and neck carcinogenesis and tumor growth, an event accompanied by increased local levels of inflammatory mediators.

## Conclusions

This study reveals for the first time that social isolation stress may increase OSCC occurrence in a pre-clinical model. Social isolation stress was also associated to an increased tumor growth, body weight loss and increased levels of inflammatory mediators in the tumor microenvironment.
